# Perspective: Solving the Heterogeneity Conundrum of TSPO PET Imaging in Psychosis

**DOI:** 10.3389/fpsyt.2020.00362

**Published:** 2020-05-01

**Authors:** Livia De Picker, Manuel Morrens

**Affiliations:** ^1^Collaborative Antwerp Psychiatric Research Institute, University of Antwerp, Antwerp, Belgium; ^2^SINAPS, University Psychiatric Hospital Campus Duffel, Duffel, Belgium

**Keywords:** translocator protein, schizophrenia, psychosis, microglia, positron emission tomography, neuroinflammation, astrocytes

## Abstract

Positron emission tomography using ligands targeting translocator protein 18 kDa (TSPO PET) is an innovative method to visualize and quantify glial inflammatory responses in the central nervous system *in vivo*. Compared to some other neuropsychiatric disorders, findings of TSPO PET in schizophrenia and related psychotic disorders have been considerably more heterogeneous. Two conflicting meta-analyses have been published on the topic within the last year: one asserting evidence for decreased TSPO uptake, while the other observed increased TSPO uptake in a selection of studies. In this paper, we review and discuss five hypotheses which may explain the observed variability of TSPO PET findings in psychotic illness, namely that (1) an inflammatory phenotype is only present in a subgroup of psychosis patients; (2) heterogeneity is caused by interference of antipsychotic medication; (3) interference of other clinical confounders in the study populations (such as age, sex, BMI, smoking, and substance use); or (4) methodological variability between studies (such as choice of tracer and kinetic model, genotyping, study power, and diurnal effects); and (5) the glial responses underlying changes in TSPO expression are themselves heterogeneous and dynamic. Finally, we propose four key recommendations for future research proposals to mitigate these different causes of heterogeneity.

## Introduction

Heterogeneity seems to come with the territory of psychiatry, and the study of positron emission tomography (PET) imaging of immune alterations in the central nervous system using nuclear ligands targeting translocator protein 18 kDA (TSPO) in psychotic disorders is no different. Ten years after the first studies were published, mixed results have remained an obstinate problem. In 2017, we published the first systematic review on TSPO PET imaging in psychotic illness (1). In that year, a series of negative studies had just emerged (2–4), bluntly sobering the initial enthusiasm that had accompanied early positive results (5, 6). Today, confusion and disappointment are tangibly present as even meta-analyses are openly contradicting each other: While Marques et al. found a significant increase in TSPO binding, based mainly on studies using first-generation TSPO tracer [11C]PK11195 (cfr. **Table 1**), Plaven-Sigray et al. found very strong evidence of decreased levels of TSPO in their meta-analysis using single-participant data of second-generation tracer studies (18, 19). To date, 14 studies have measured TSPO tracer binding in schizophrenia-spectrum disorders, of which seven were conducted in patients within the first 5 years of diagnosis (cfr. **Table 1**). Three studies included at ultra-high risk for psychosis (2, 4, 10). We have previously highlighted variability in terms of study population, tracer, kinetic modeling, and outcome measures (1). In this paper, we will discuss five potential sources of heterogeneity in TSPO PET imaging in psychotic illness.

**Table 1:** PET studies with TSPO tracer in patients with psychotic illness *versus* controls.

1A. Original studies									
Author (year)	n P	n C	Tracer	Model; Outcome measure	Clinical state *PANSS Total (T) and positive (P) symptom scale score*	Mean age P	% male P	% medicated P	Outcome
van Berckel et al. (6)	10	10	[11C]PK11195	2TCM; BP	Undefined Symptom scores unavailable	24 ± 2	90%	100%	SZ **>** C
Doorduin et al. (5)	7	8	[11C]PK11195	2TCM; BP	Psychosis *T 73.6 ± 13.3* *P 19.7 ± 3.0*	31.2 ± 7.2	85.7%	100%	SZ **>** C
Banati and Hickie (7)	16	8	[11C]PK11195	2TCM; BP	Undefined				SZ **>** C
Takano et al. (8)	14	14	[11C]DAA1106	2TCM; BP	Chronic *T 77.9 ± 20.1* *P 19.1 ± 5.3*	43.8 ± 7.4	57.1%	100%	SZ=C
Kenk et al. (9)	16	27	[18F]FEPPA	2TCM; V_T_	Psychosis *T 70.2 ± 9.7* *P 19.3 ± 2.2*	42.5 ± 14.0	62.5%	100%	SZ=C
Bloomfield et al. (10)	14	14	[11C]PBR28	2TCM-1K; DVR	Undefined *T 63.7 ± 18.1* *P 17.0 ± 6.1*	47.0 ± 9.3	75%	100%	SZ **>** C
Coughlin et al. (11)	12	14	[11C]DPA713	Undefined; V_T_	Undefined *T unavailable* *P (SAPS) 3.8 ± 2.5*	24.3 ± 3.3	75%	83%	SZ=C
Holmes et al. (12)	16	16	[11C]PK11195	Reference tissue; BP	Undefined	33 ± 9	68.8%	50%	SZ > C if medicated
Van der Doef, 2016 (3)	19	17	[11C]PK11195	Reference tissue; BP	Undefined *T 53 ± 10* *P 12 ± 4*	26 ± 4	84.2%	79%	SZ=C
Collste et al. (13)	16	16	[11C]PBR28	2TCM; V_T_	FEP drug naïve *T 77.4 ± 18.3* *P 20.3 ± 4.9*	28.5 ± 8.4	68.8%	0%	SZ **<** C
Hafizi et al. (14)	19	20	[18F]FEPPA	2TCM; V_T_	FEP unmedicated *T 68.6 ± 13.0* *P 19.2 ± 3.8*	27.5 ± 6.8	63.2%	0%	SZ=C
Di Biase et al. (2)	33	27	[11C]PK11195	Reference tissue; BP	Recent-onset (n=18)				
					T 68.5* P (BPRS) *12.6 ± 4.6*	20.6 ± 5.5	88.9%	78%	SZ=C
					Chronic (n=15) T 86.5* P (BPRS) *19.5 ± 7.8*	35.2 ± 6.6	66.7%	100%	SZ=C
Ottoy et al. (15) & De Picker et al. (16)	14	17	[18F]PBR111	2TCM-1K; V_T_	Acute psychosis & remission *T 75.3 ± 21.0* *P 24.1 ± 5.4*	32.2 ± 8.3	100%	90%	SZ > C if P > 30y
Laurikainen et al. (17)	14	15	[11C]PBR28	2TCM; V_T_	FEP *T (BPRS-E) 60 ± 18*	24.8 ± 4	63.6%	87.6%	SZ < C
**1B. META-ANALYSES**									
Author (year)	nP	nC	n studies	Model; Outcome measure	Clinical state	Mean age P	% male P	% medicated	Outcome
Marques et al. (18)	190	200	6 (5/6 [11C]PK11195) 6 (second-generation)	BP V_T_					SZ > C g=0.31 SZ=C g=-0.22
Plaven-Sigray et al. (19)	75	77	5 (second-generation)	2TCM; V_T_	*P 18.2 ± 4.2*	33.9 ± 12.6	68%	52%	SZ < C SMD=0.47-0.63

SZ, schizophrenia patient group; C, control group; n, number of subjects; FEP, first episode psychosis patients; “DOI”, duration of illness; 2TCM, two-tissue compartment model, BP, binding potential; V_T_, volume of distribution; DVR, distribution volume ratio; CPZ, chlorpromazine equivalent; SZ > C, increased uptake of tracer in schizophrenia patients compared to controls; SZ=C, no difference in tracer uptake between schizophrenia patients and controls; SZ < C, decreased uptake of tracer in schizophrenia patients compared to controls.

*mean BPRS total scores were converted to corresponding PANSS total scores using the equipercentile linking method (20).

TSPO PET results in ultra-high risk patient (sub)samples are not included in the table.

## Hypothesis 1: An Inflammatory Phenotype is Present in a Subgroup of Schizophrenia Patients

It has been proposed that one or more different “immunophenotypes” (i.e., immune-inflammatory biotypes) may exist within the psychotic population (21). While this hypothesis has not been studied within the central nervous system, a recently published meta-analysis of 35 studies of peripheral immune markers among first-episode psychosis did not point towards the existence of subgroups (22). However, pro-inflammatory cytokines IL-6 and IFN-γ are elevated in first-episode patients who do not respond to antipsychotic treatment relative to those who do respond, raising the possibility that this treatment-resistant subgroup, could involve specific CNS immune changes (23). This hypothesis has yet to be tested with TSPO PET imaging.

## Hypothesis 2: Heterogeneity Through Interference of Medication Status

For obvious ethical and practical reasons, most patients suffering from psychotic illness will be or have previously been exposed to antipsychotic medication. Antipsychotics are known to differentially affect microglial activation (increased with haloperidol and olanzapine, but reduced with risperidone) (24, 25) and TSPO binding (increased with clozapine, no significant changes with sulpiride) in rats (26). The interpretation of TSPO PET results in schizophrenia is therefore confounded by a medication effect of unknown size. Prior to the use of TSPO PET, two [3H]PK11195 autoradiography studies have found the number of TSPO binding sites in peripheral platelet cells to demonstrate a 30% decrease in patients who had been (chronically) medicated with antipsychotics relative to unmedicated patients and age-matched controls (27–29). This contradicts findings of two [11C]PK11195 PET studies who found that unmedicated patients had relatively lower TSPO levels compared with antipsychotic-treated patients (2, 12). However, as the TSPO imaging in these unmedicated psychotic patients took place very early in the illness course, during the prodromal stage or early in the first psychotic symptoms while the medicated patients were older and had a longer duration of illness, they cannot differentiate between medication and dynamic effects of illness state and progression (16). Plaven-Sigray et al. did not find a difference in TSPO levels between drug-free and medicated patients in their meta-analysis (19).

## Hypothesis 3: Heterogeneity Through Interference of Other Clinical Confounders

### Age, Sex, and BMI Effects

**Figure 1:**
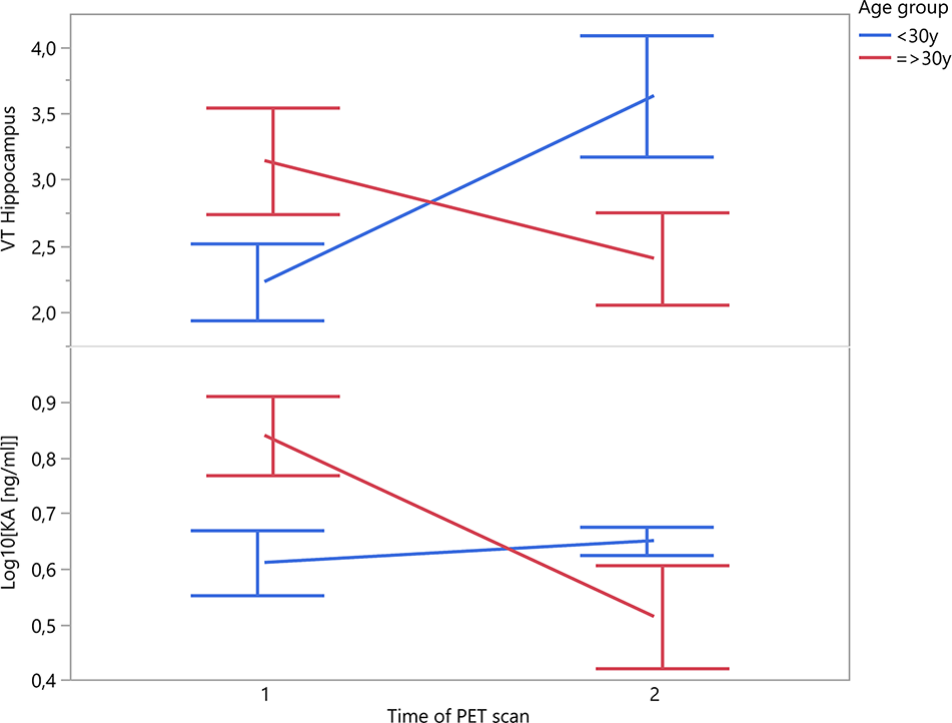
Patients’ change in TSPO binding in hippocampal region and plasma KA over the time course of a psychotic episode (n = 10; time interval 12 ± 4 weeks).

A recent multicentric study confirmed significant positive correlations between age and TSPO binding in the frontal and temporal cortex of 140 healthy volunteers, and a significant positive correlation with age in all brain regions in male subjects (30). It is therefore very likely that age may also have an impact on TSPO binding in disease states. In our own work, a significant interaction with age was found to influence the TSPO binding of patients over the longitudinal course of a psychotic episode (cfr. **Figure 1**) (15, 16). While such a dynamic interaction with age offers a compelling explanation for some of the discrepancies between different study cohorts of psychosis patients, this finding deserves independent replication before drawing firm conclusions.

Not unimportantly in the predominantly male schizophrenia patient group, the same multicentric study has indicated that females show significantly higher TSPO binding in all regions compared to males. TSPO mediates the rate-limiting step of steroidogenesis. An alteration in TSPO levels could thus affect the production of neurosteroids in the brain, among which female sex hormones, independently of peripheral endocrine sources (31). Finally, many patients—especially those with longer duration of illness and exposure to atypical antipsychotics—also tend towards higher BMIs, which correlate inversely with TSPO uptake (30).

### Smoking and Substance Use Effects

In a cohort of healthy controls, smokers had a 15.5–17.0% decreased TSPO uptake, both in the satiated state and after a night of abstention, compared to nonsmokers (32, 33). If chronic cigarette smoking leads to a global reduction in TSPO binding, this could markedly influence the results of cohorts of psychotic patients, known to be active smokers at a much higher prevalence than the general population (32, 33). A similar effect is true for cannabis use, which is also highly prevalent among schizophrenia patients. Most studies of TSPO PET in schizophrenia will include patients that have a history of cannabis use if they remain abstinent during their study participation and/or are inpatients with restricted access to illicit substances. A cohort of long-term cannabis users (using cannabis at least 4 times per week for 12 months and/or meeting criteria for cannabis use disorder) had a 23.3% increased TSPO binding compared to non-users (34). Given the long biological half-life of cannabinoids, these effects may very well persist into abstinence, although this has not yet been studied. It is unclear what the effect would be of concomitant use of nicotine and cannabis products, and if vaping of nicotine- and/or THC-containing liquids exerts the same effect. In our own work, smoking status and cannabis use were not identified as significant confounders; however, the unequal distribution of (nicotine and/or cannabis) smokers between cohorts could mask such an effect.

Two studies of TSPO PET in alcohol use disorder patients have demonstrated 10–20% decreased TSPO binding in recently detoxified patients (35, 36). Intriguingly, increased binding was shown with acute alcohol exposure in a non-human primate “binge drinking” model—persisting for several months after abstinence (37). Abuse of methamphetamine (but not cocaine) has also been demonstrated to significantly affect TSPO binding (38, 39). Ideally, future studies should aim to match cohorts for smoking status, as well as current and prior substance use.

## Hypothesis 4: Heterogeneity Through Methodological Variations

### Choice of TSPO Tracer and Kinetic Modeling

A major limitation of TSPO PET imaging studies is that the quantification of data is a complex task. Methodological issues related to this process have been extensively discussed by other authors (19, 40–44). In summary, because microglia are distributed ubiquitously throughout the entire brain, a traditional reference tissue approach is problematic as no brain region of interest can be relied upon to be devoid of specific signal (45). Advanced methods using cluster analysis have been devised to determine a suitable reference region (43). Until these methods have been thoroughly validated for multiple ligands, an arterial input function remains the gold standard to reliably quantify the TSPO radioligand signal. A concern is that (radial) arterial sampling is invasive and arduous to patients, leading to higher inclusion bias and drop-out rates. A total of six different TSPO tracers have been applied in the clinical study of schizophrenia/psychotic illness. The advantages and disadvantages as well as the specific sensitivity of each tracer to TSPO protein have been reviewed elsewhere (46, 47). First-generation TSPO radiotracers, such as [11C]PK11195, exhibit poor extraction and lower signal-to-noise than second-generation tracers (e.g., [11C]PBR28, [11C]DPA-713, [18F]PBR111, or [18F]FEPPA) and tend to use outcome measure non-displaceable binding (BP: tracer binding in region of interest relative to other “reference” brain regions) instead of “gold standard” outcome measure volume of distribution (V_T_: total amount of tracer in region of interest relative to blood). Marques et al. found a significant increased TSPO level with studies using BP as outcome measure (with five out of six studies using first-generation tracer [11C]PK11195) (18), but the methodological validity of these findings are controversial (44)—recent findings suggest that at least half of the variability in [11C]PK11195 studies is due to measurement error (44). Other sources of methodological variation and potential bias are the correction for plasma free fraction and ligand binding to plasma proteins such as α1-acid glycoprotein, variability in outcome measurements, the inclusion of an additional endothelial compartment to the traditional two-compartment model (13, 27, 42).

### Genotyping

In 2010, Owen et al. warned that second-generation ligand [11C]PBR28 does not produce a specific binding signal in approximately 14% of healthy volunteers (48). One year later, they demonstrated that all second-generation TSPO ligands in clinical use recognize high affinity (HABs, 66% of the Caucasian population), mixed affinity (MABs, 29%), and low affinity binders (LABs, 5%) in brain tissue *in vitro* (49). A single nucleotide polymorphism (rs6971) in exon 4 of the TSPO gene causes an alanine-to-threonine substitution affecting the ligand-binding affinity of TSPO. Prior genotyping of subjects for this polymorphism (with exclusion of LAB and stratification between MAB and HAB) is therefore required to reliably quantify TSPO binding with second-generation radiotracers. One Japanese study by Takano et al. (8) was published before this knowledge became available and therefore failed to correct for this important confounder yet the prevalence of the low-binding allele is estimated at only 4% in this demographic (8, 50). Some TSPO studies in psychotic illness have reported significant findings in one genotype group, but not in the other, thereby confusing overall interpretation. For instance, Marques et al. found no significant difference in HAB, but a significant decrease of TSPO binding in MAB subjects with second-generation tracers (18).

### Sample Size/Study Power

The relatively small patient sample sizes in TSPO PET studies of schizophrenia patients have often been criticized (44). Yet the mean sample size in TSPO PET studies across 41 neuropsychiatric diagnoses is not significantly different from studies in schizophrenia/psychotic illness (17.4 ± 10.9 all diagnoses versus 19.9 ± 9.0 in psychotic illness; De Picker et al., in prep). However, as mentioned above, in second-generation TSPO tracers’ stratification of study groups by genotype is required. To compensate for this loss of study power, the sample size in studies of second-generation TSPO tracers has been on average 46% larger than with [11C]PK11195 (12.7 ± 1.9 vs. 19.0 ± 1.4) across diagnostic categories, but only 10% larger in studies of psychotic illness (18.4 ± 3.4 vs. 20.3 ± 2.7; De Picker et al., in prep). As most of the studies using second-generation tracers were published in the last 5 years, at an average study completion time of 4–5 years, power calculations have probably been based on the effect size estimates of the earlier [11C]PK11195 studies (published in 2008–2009), which in retrospect may have reported inflated effect sizes (44). We therefore cannot exclude the possibility that some of the later second-generation ligand studies have been underpowered.

### Diurnal Effect

Specific immune cells and cytokines show a 24-hour circadian variation in plasma and CSF—similar diurnal changes may also exist in TSPO binding (51). A 18.5 ± 23.9% higher V_T_ was observed in grey matter of healthy subjects in the afternoon compared to the morning of the same day (52).

## Hypothesis 5: Glial Responses Underlying TSPO Changes are Heterogeneous and Dynamic

TSPO is expressed at low levels at the outer mitochondrial membrane of various cell types, including microglia, astrocytes, and vascular endothelial cells throughout the brain and increases sharply in response to neuronal injury and inflammation. TSPO is often considered a biomarker of “neuroinflammation” or “microglial activation”, yet novel findings have indicated this notion is erroneous and it is more appropriate to equate TSPO binding to glial responses in general. Firstly, “neuroinflammation” is essentially a spectrum of still ill-defined physiological functions and dynamic response patterns which varies with the type and course of a pathological condition. Contingent upon the integrity of the blood-brain barrier (BBB) and a condition’s regional focus, distinct patterns of TSPO upregulation can ensue in different brain pathologies. Secondly, our knowledge on the cellular mechanisms of neuroinflammation is suboptimal (1). Studies in animal models have compellingly demonstrated the increased TSPO signal in brain pathology is derived from both microglial cells and astrocytes, in a dynamic temporal interplay (summarized by Guilarte, 2019) (27). Following exposure to a neurotoxic substance, an early microglial response at 2 weeks is followed by a later astrocytic activation and further increase in TSPO levels at 3–4 weeks. Upon removal of the toxic compound, the TSPO signal gradually decreases (50% decrease after 6 weeks), with the astrocytic signal enduring after the microglial response has already subsided (27). It is also largely unknown how central and peripheral inflammatory responses cross-talk with each other. TSPO levels have been demonstrated to increase 30% within 1 hour and 60% after 4 hours following a classical immune challenge, correlating with an increase in blood levels of inflammatory cytokines as well as sickness symptoms (53, 54). Yet in some auto-immune conditions, increased peripheral cytokines were found to be inversely correlated with TSPO binding. Likewise, reduced prefrontal TSPO levels were found in an infection-mediated neurodevelopmental mouse model, accompanied with increases in inflammatory cytokines and schizophrenia-relevant behavioral abnormalities (55).

Given the considerable intra- and inter-individual variability in symptomatology, treatment response and illness course among patients with psychotic disorders, cross-sectional studies clearly do not provide an accurate representation of the dynamic nature of glial responses. TSPO levels in psychotic illness could be differentially altered in specific symptomatic states (i.e., acute psychotic syndrome, negative symptoms) or stages throughout the illness course (i.e., prodromal, relapsing-remitting, chronic, and treatment-resistant), depending on the differential recruitment from different cellular sources. Both microglia and astrocytes have been implicated in post-mortem research of schizophrenia patients. Kynurenic acid (KA), an astrocyte-derived neuroinhibitory tryptophan neurometabolite, is at the core of the hypothesis linking neuroinflammation to psychotic illness. KA reduces striatal extracellular dopamine through antagonism of α_7_nAch- and NMDA receptors (56). Increased central and decreased peripheral levels of KA have been found in schizophrenia (57). In our own work, the evolution of the TSPO expression over the course of a psychotic episode differed in subjects under the age of 30 compared to those who were older (mean scan interval 12.3 ± 4.6 weeks) (16). Interestingly, in these same subjects, plasma KA levels mimicked this effect (cfr **Figure 1**; unpublished data). Even if these findings only concern a relatively small sample size of n = 10 patients, they corroborate the interesting question whether dynamic TSPO changes could be related to a differential recruitment of microglial and astrocytic populations in an age-dependent or illness-specific pattern.

Finally, regardless of the cellular source, TSPO is not functionally involved in neuroimmune signaling and therefore also does not reliably identify pro- versus anti-inflammatory processes. A consistent downregulation of TSPO emerged in macrophages activated to a pro-inflammatory, or “M1” phenotype. Conversely, stimulation of macrophages to an “M2” phenotype with IL-4, dexamethasone or TGF-β1 did not alter TSPO expression (58). However, findings derived from the study of macrophages or rodent microglia cannot be reliably extrapolated to the behavior of human microglial cells *in vivo* (59, 60). Even in those neuroinflammatory conditions accompanied by clear and unequivocal TSPO upregulation, better understanding of the functional meaning of these glial responses is crucial before we can jump to therapeutic avenues. It has been proposed that because neuroinflammation plays a central role in the progression of neurodegenerative diseases, drugs such as minocycline and cyclooxygenase (COX) inhibitors could be beneficial for their *in vivo* anti-inflammatory—hence neuroprotective—properties. The underlying assumption that increased glial responses are pathological and detrimental to the brain is however simply not true. This has been painfully demonstrated in a clinical trial of 15 patients who had suffered moderate-to-severe traumatic brain injury, randomized to receive either minocycline 200 mg per day or no drug for 12 weeks. While minocycline effectively reduced the glial activity on TSPO PET, it also increased markers of neurodegeneration (61). It appears in the specific case of traumatic brain injury the observed glial responses are of a reparative rather than a pathological nature. On the other hand, both minocycline and COX-2 inhibitor celecoxib have been shown to be clinically beneficial when used as add-on treatment to antipsychotics in psychotic patients, even though the TSPO PET results in psychotic patients have been ambivalent (1).

## Discussion and Recommendations

Even at the level of simple nosology, clinicians and researchers in psychotic disorders understand that diagnostic categories do not represent valid underlying constructs and are more likely to encompass a wider range of disease entities or subgroups, emerging as a similar clinical syndrome. It therefore does not come as a surprise that heterogeneity also springs up in the research on its underlying neurobiology. In fact, as has recently been demonstrated by Brugger et al. in their meta-analyses of intra-individual variance of regional brain structure (62) and PET imaging of striatal dopaminergic transmission (63), this heterogeneity does not have to be an insurmountable challenge, and can instead be turned into an interesting research question in and of itself. Importantly, Plaven-Sigray et al. have noted that, when all clinical and technical confounders were accounted for, the heterogeneity between studies in their meta-analysis was actually quite low (19). This highlights the importance of using a suitable study design which serves to minimize the variability caused by methodological problems. Still, the emergence of heterogeneous results in the study of TSPO in psychotic illness over the last 5 years has paradoxically raised new questions about the nature and the dynamics of the underlying glial responses and how they relate to TSPO binding in schizophrenia and other illnesses.

Compared to other neuroinflammatory conditions, psychotic illnesses come with some built-in disadvantages. They cannot rely as much on animal or other preclinical models to translationally evaluate the role of neuroinflammatory and glial mechanisms. There is no consistent illness-free region which could serve as reference in imaging studies, and confounding effects of medication and substance use are difficult to eradicate. Furthermore, because the underlying pathophysiology of schizophrenia—specifically, the sequence of causal events in the development of the disorder—is largely unknown, immune and glial mechanisms could be subject to disease-specific, state-specific, and age-specific alterations. Given the considerable cost and effort involved in executing these studies, often taking 4 to 5 years to complete, research groups as well as funding agencies may be reluctant to invest in the field of TSPO PET imaging in psychotic illness any further until we find a way to obtain more consistent results. It is very likely that the true cause of the observed heterogeneity is multifactorial in nature, with several or all of the abovementioned hypotheses contributing to some extent. We therefore advocate future research proposals to take into consideration the following recommendations to mitigate the different reasons for heterogeneity:

1. A consensus on the optimal methodology for TSPO PET imaging in psychotic disorders, which can be applied and replicated in further studies, needs to be reached. Because of the low reliability and sensitivity of (R)-[11C]PK11195 outcomes, preference should be given to second-generation tracers with arterial input function kinetic modeling. In terms of reporting, we advocate for adherence to the guidelines on the content and format of PET brain data publications which were recently provided in a consensus paper (64). Specifically for TSPO PET, both 2TCM and 2TCM-1K V_T_ outcome measures should be reported. Cohorts should be genotyped for rs6971 and power calculations should establish adequate sample sizes for each genotype subgroup;
2. Control cohorts should be matched for age, sex, BMI, smoking status, and prior substance use (up to 3 months before PET scan) as well as genotype and time of scan. If matching for substance use is not possible, a careful history of any substance use in the last 3 months prior to the scan needs to be reported;
3. Longitudinal studies are needed to track the evolution of TSPO expression changes through different phases of the illness, as well as before and after the initiation of antipsychotic medication;
4. Studies should stratify patients according to relevant subgroups such as immunophenotypes, treatment-resistant or ultra-high-risk individuals, and different age groups. Findings should be corrected for (lifetime) cumulative medication exposure.

## Data Availability Statement

The datasets generated for this study are available on request to the corresponding author.

## Author Contributions

LP devised the main conceptual ideas and proof outline. MM aided in interpreting the results and deciding on the final scope of the manuscript. LP wrote the manuscript in consultation with MM.

## Funding

The authors have received funding from University Hospital Duffel/University of Antwerp to cover open access publication fees.

## Conflict of Interest

The authors declare that the research was conducted in the absence of any commercial or financial relationships that could be construed as a potential conflict of interest.
